# *In Vitro* and *In Vivo* Activity of Peptidomimetic Compounds That Target the Periodontal Pathogen Porphyromonas gingivalis

**DOI:** 10.1128/AAC.00400-18

**Published:** 2018-06-26

**Authors:** Jinlian Tan, Pravin C. Patil, Frederick A. Luzzio, Donald R. Demuth

**Affiliations:** a Department of Oral Immunology and Infectious Diseases, University of Louisville School of Dentistry, Louisville, Kentucky, USA; b Department of Chemistry, University of Louisville College of Arts and Sciences, Louisville, Kentucky, USA

**Keywords:** Porphyromonas*gingivalis*, peptidomimetic, periodontitis

## Abstract

The interaction of the periodontal pathogen Porphyromonas gingivalis with oral streptococci is important for initial colonization of the oral cavity by P. gingivalis and is mediated by a discrete motif of the streptococcal antigen I/II protein. A synthetic peptide encompassing this motif functions as a potent inhibitor of P. gingivalis adherence, but the use of peptides as topically applied therapeutic agents in the oral cavity has limitations arising from the relatively high cost of peptide synthesis and their susceptibility to degradation by proteases expressed by oral organisms. In this study, we demonstrate the *in vitro* and *in vivo* activity of five small-molecule mimetic compounds of the streptococcal peptide. Using a three-species biofilm model, all five compounds were shown to effectively inhibit the incorporation of P. gingivalis into *in vitro* biofilms and exhibited 50% inhibitory concentrations (IC_50_s) of 10 to 20 μM. Four of the five compounds also significantly reduced maxillary alveolar bone resorption induced by P. gingivalis infection in a mouse model of periodontitis. All of the compounds were nontoxic toward a human telomerase immortalized gingival keratinocyte cell line. Three compounds exhibited slight toxicity against the murine macrophage J774A.1 cell line at the highest concentration tested. Compound PCP-III-201 was nontoxic to both cell lines and the most potent inhibitor of P. gingivalis virulence and thus may represent a novel potential therapeutic agent that targets P. gingivalis by preventing its colonization of the oral cavity.

## INTRODUCTION

Periodontitis is a widespread inflammatory disease that is caused by a consortium of anaerobic bacteria, including Porphyromonas gingivalis, Tannerella forsythia, and Treponema denticola (1–5). Of these organisms, P. gingivalis has been the most extensively studied and has been suggested to represent a keystone pathogen that targets the host innate immune response, leading to disruption of normal host-microbe homeostasis (dysbiosis). This results in increased microbial biomass and significant population shifts in the oral microbiome, leading to chronic inflammation (6–8). Periodontitis has also been associated with a variety of systemic disorders such as cardiovascular disease, diabetes mellitus, and rheumatoid arthritis (9–12). Current methods to treat periodontitis involve removing the microbial biofilm by scaling and root planning, and in more severe cases, surgery may be required to reduce gingival pocket depth. In general, therapeutic approaches that specifically target periodontal pathogens like P. gingivalis are lacking, and methods that prevent or limit the recolonization of the oral cavity by P. gingivalis after clinical treatment of diseased sites are not available. Thus, the disease commonly recurs and requires additional treatment.

The primary niche for P. gingivalis is in a mixed community of bacterial species that reside in the subgingival pocket; however, upon initial entry into the oral cavity, P. gingivalis initially colonizes supragingival plaque and interacts with oral streptococci (13). Our previous results suggested that the interaction of P. gingivalis with oral streptococci is important for this early colonization event (14, 15) and thus represents an ideal point for therapeutic intervention to control the initial colonization or recolonization of oral tissues by P. gingivalis. Adherence of P. gingivalis to streptococci is species specific and is driven by a protein-protein interaction that occurs between the minor fimbrial antigen (Mfa) of P. gingivalis and the antigen I/II (Ag I/II) polypeptide of streptococci (16–18). Daep et al. identified a discrete domain in Ag I/II protein that mediates its interaction with Mfa and showed that this region resembles the eukaryotic nuclear receptor (NR) box protein-protein interaction domain (16, 17). Within the NR box-like domain are two functional peptide motifs, VXXLL and NITVK, that are essential for P. gingivalis adherence to streptococci. Daep et al. also showed that a synthetic peptide encompassing both motifs functioned as a potent inhibitor of P. gingivalis adherence and significantly reduced P. gingivalis virulence *in vivo* (17, 18). These studies suggest that P. gingivalis colonization of the oral cavity may be controlled by preventing its initial association with streptococci and that inhibitors of the Mfa-Ag I/II interaction represent potential therapeutic agents to treat or prevent recurrence of periodontitis.

The use of peptides as topically applied therapeutic agents in the oral cavity has limitations arising from the relatively high cost of peptide synthesis and their susceptibility to degradation by proteases expressed by oral organisms, including P. gingivalis itself. To address these limitations, Patil et al. (19) designed and synthesized potent and stable small-molecule inhibitors that mimic the natural peptide substrate recognized by Mfa by employing a strategy that joined mimics of VXXLL and NITVK together via the “click” reaction (20, 21). Within the expansive area of nitrogen/oxygen heterocycles, the 2,4,5-trisubstituted oxazole framework was selected as a starting point for the NITVK-associated inhibitors of Mfa-Ag I/II interaction (22, 23), and several of these compounds potently blocked P. gingivalis adherence to streptococci *in vitro* when click coupled with substituted arylalkynes. In this study, we show that five small-molecule mimetic compounds inhibit the incorporation of P. gingivalis into a microbial biofilm and reduce P. gingivalis virulence in a mouse model of periodontitis when administered simultaneously with P. gingivalis oral infection. The most potent compounds do not exhibit significant toxicity against human gingival epithelial or mouse macrophage cell lines at concentrations that inhibit biofilm formation or virulence and thus may represent novel therapeutics to limit P. gingivalis colonization of the oral cavity.

## RESULTS

### Inhibition of P. gingivalis biofilm formation.

**FIG 1:**
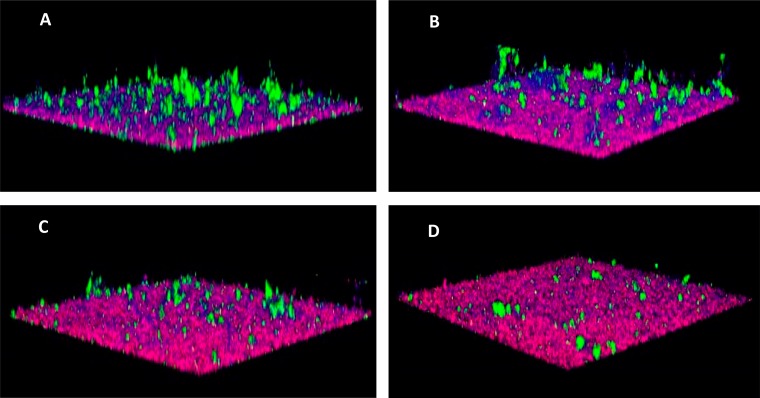
Representative images of a three-species biofilm comprising S. gordonii (red), F. nucleatum (blue), and P. gingivalis (green). (A) Control biofilm formed in buffer alone; (B to D) biofilms formed in buffer containing 5 μM, 10 μM, or 20 μM PCP-III-201, respectively.

Fifty peptidomimetic compounds were previously tested for inhibition of P. gingivalis adherence to Streptococcus gordonii (19), and the five most potent inhibitors were selected for further analysis in this study. The 50% inhibitory concentrations (IC_50_s) for inhibition of interspecies adherence by these compounds ranged from 5 to 15 μM (19). In the oral cavity, however, the microbiome is more complex, and P. gingivalis and S. gordonii form biofilms in the presence of other bridging organisms that can independently adhere to both P. gingivalis and S. gordonii. To determine whether the peptidomimetic compounds inhibit P. gingivalis biofilm formation in the presence of a bridging organism, a three-species biofilm model was employed in which P. gingivalis and S. gordonii were incubated in the presence of a bridging organism, Fusobacterium nucleatum. As shown in Table 1, all five compounds inhibited the incorporation of P. gingivalis into the three-species biofilm and exhibited IC_50_s between 10 and 20 μM, similar to the IC_50_s previously reported for inhibition of P. gingivalis adherence to streptococci (19). A representative image showing the three-species biofilms obtained in the presence of compound PCP-III-201 is shown in Fig. 1. Interestingly, in addition to reducing the levels of P. gingivalis, each compound also reduced the level of F. nucleatum adhered to streptococci. Thus, the peptidomimetic compounds prevented the interaction of P. gingivalis with S. gordonii and inhibited biofilm formation in the presence of a bridging species, suggesting that the compounds may be effective in reducing P. gingivalis colonization of more-complex microbial communities.

**TABLE 1:**
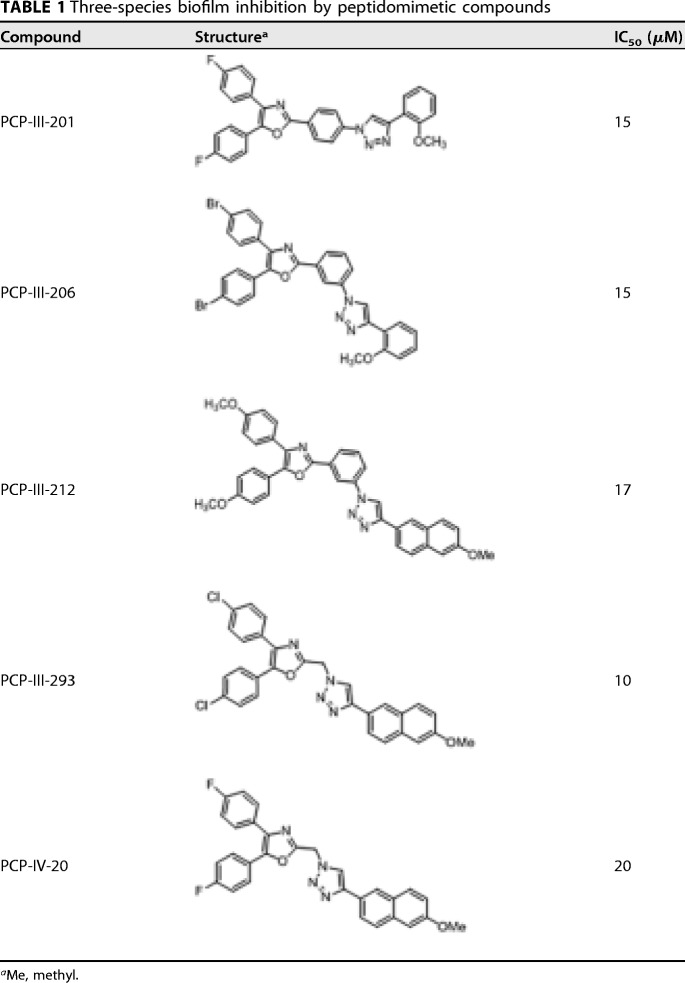
Three-species biofilm inhibition by peptidomimetic compounds

a Me, methyl.

Compound PCP-III-201 was also tested for activity against a preformed three-species biofilm. Representative images of three-species biofilms treated with PCP-III-201 are shown in Fig. S1 in the supplemental material. As shown in Fig. S1 and Table 2, a dose- and time-dependent inhibition was observed. This indicates that in addition to inhibiting the formation of a three-species biofilm, the compound was able to disrupt a preformed biofilm.

**TABLE 2:** Activity of PCP-III-201 against a preformed biofilm

Treatment time (h)	% inhibition with the indicated concn of PCP-III-201		
	5 μM	10 μM	20 μM
1	3.3	22.3	49.5
2	2.5	35.4	58.7
3	29.5	41.4	60.5

### Inhibition of P. gingivalis virulence by peptidomimetic compounds.

**FIG 2:**
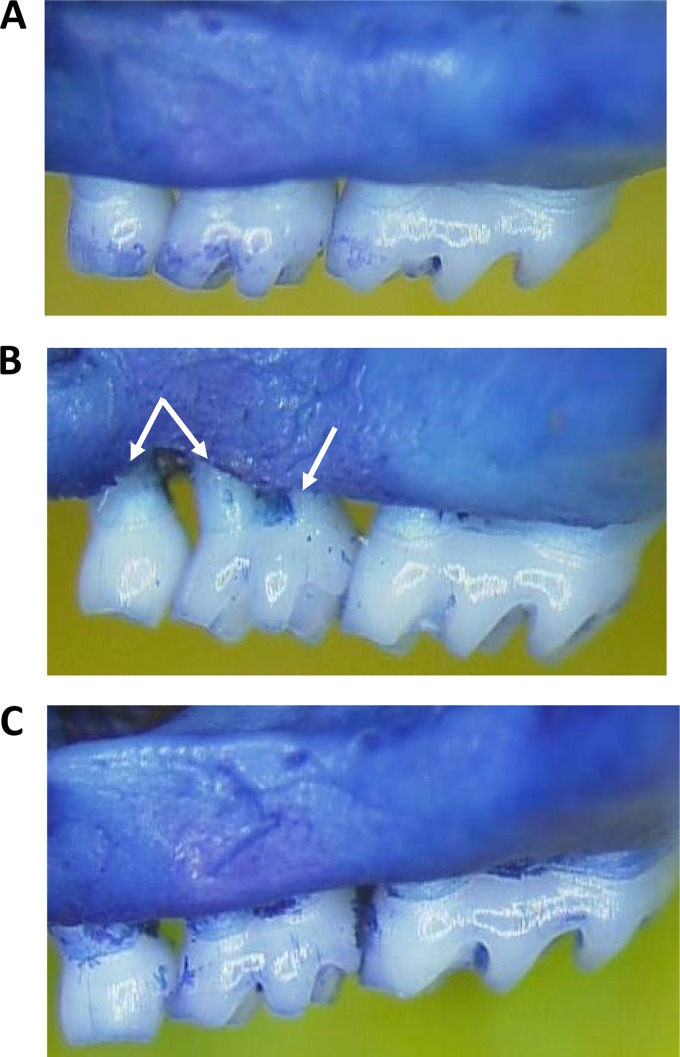
Representative images of the maxillary molars of sham-infected animals (A), P. gingivalis-infected animals (B), and animals infected with P. gingivalis in the presence of 20 μM PCP-III-201 (C).

**FIG 3:**
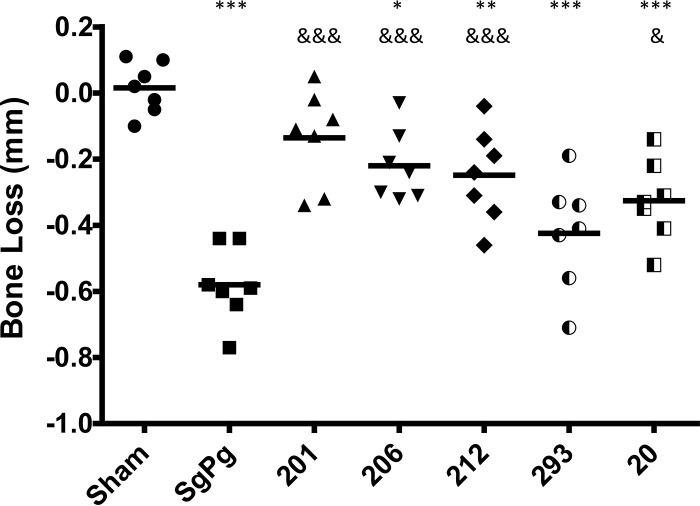
Alveolar bone loss of untreated P. gingivalis-infected mice (SgPg) and animals infected with P. gingivalis in the presence of peptidomimetic compound PCP-III-201 (201), PCP-III-206, PCP-III-212, PCP-III-293, or PCP-IV-20. Each symbol represents the value for an individual animal. The black bars are the mean values for the groups. Asterisks indicate a significant increase in bone loss relative to sham-infected animals as follows; *, *P* < 0.05; **, *P* < 0.01; ***, *P* < 0.001. Ampersands indicate a significant reduction in bone loss relative to untreated mice (group SgPg) as follows; &, *P* < 0.05; &&&, *P* < 0.001.

Next, to determine whether the peptidomimetic compounds affect P. gingivalis virulence *in vivo*, each compound was examined in a mouse model of periodontitis that was previously described by Daep et al. (18). Since a primary clinical outcome of periodontitis in humans is the resorption of alveolar bone supporting the teeth, virulence was assessed in this model by measuring the extent of alveolar bone loss around the maxillary molars that was induced by P. gingivalis infection. Representative images of the murine maxilla obtained from sham-infected, P. gingivalis-infected, and treated animals are shown in Fig. 2. Sham-infected (Fig. 2A) and PCP-III-201-treated (Fig. 2C) animals exhibited a smooth alveolar bone crest (ABC), whereas P. gingivalis-infected animals (Fig. 2B) exhibited significantly increased alveolar bone resorption and an irregular ABC (indicated by arrows). Quantification of alveolar bone loss for all animal groups is shown in Fig. 3. After establishing S. gordonii in the oral cavity of mice, infection of animals with P. gingivalis (group SgPg) induced a significant increase in alveolar bone loss relative to sham-infected animals (*P* < 0.001). In contrast, infection of mice with P. gingivalis in the presence of four of the five peptidomimetic compounds significantly reduced the amount of alveolar bone loss relative to P. gingivalis-infected animals without compound (i.e., group SgPg). For mice treated with compound PCP-III-201, the extent of bone loss observed was not significantly different from sham-infected animals (*P* < 0.10). Although compounds PCP-III-206, PCP-III-212, and PCP-IV-20 significantly reduced bone loss relative to untreated P. gingivalis-infected animals (*P* < 0.01), the mice in these groups exhibited more bone loss than sham-infected animals (*P* < 0.05). The extent of bone loss after treatment with compound PCP-III-293 was not significantly reduced relative to the SgPg group (*P* < 0.16). Thus, four of the five compounds tested inhibited or partially inhibited P. gingivalis virulence.

### Toxicity of peptidomimetic compounds.

**FIG 4:**
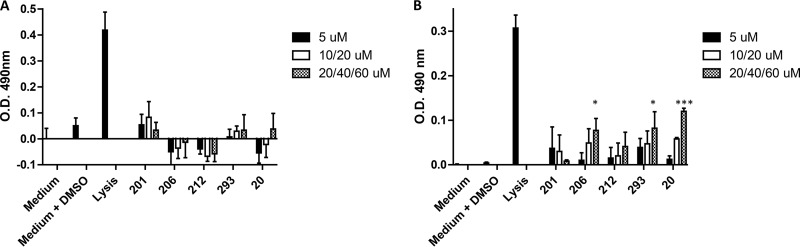
Release of lactate dehydrogenase (LDH) activity from TIGK (A) and J774A.1 (B) cells after 18-h exposure to peptidomimetic compounds. The peptidomimetic compounds and concentrations used are as follows: PCP-III-201, 5 μM, 10 μM, and 20 μM; PCP-III-206, 5 μM, 20 μM, and 40 μM; PCP-III-212, 5 μM, 10 μM, and 20 μM; PCP-III-293, 5 μM, 20 μM, and 60 μM; PCP-IV-20, 5 μM, 20 μM, and 60 μM. Asterisks indicate a significant increase in LDH activity relative to cells exposed to medium containing 0.1% DMSO as follows; *, *P* < 0.05; ***, *P* < 0.001.

**FIG 5:**
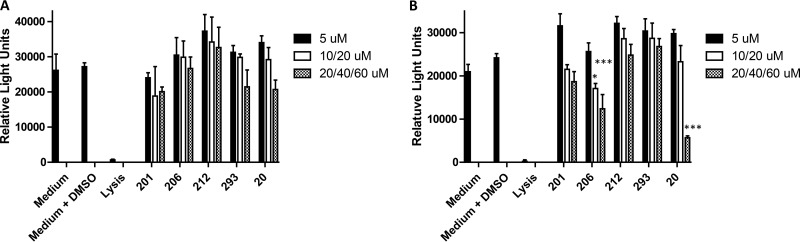
Quantification of ATP levels in TIGK (A) and J774A.1 (B) cells after 18-h exposure to peptidomimetic compounds. The peptidomimetic compounds and concentrations used are as follows: PCP-III-201, 5 μM, 10 μM, and 20 μM; PCP-III-206, 5 μM, 20 μM, and 40 μM; PCP-III-212, 5 μM, 10 μM, and 20 μM; PCP-III-293, 5 μM, 20 μM, and 60 μM; PCP-IV-20, 5 μM, 20 μM, and 60 μM. Asterisks indicate a significant decrease in ATP relative to cells exposed to medium containing 0.1% DMSO as follows; *, *P* < 0.05; ***, *P* < 0.001.

Although none of the mice exhibited visible detrimental effects after administration of the peptidomimetic compounds, four additional *in vitro* approaches were employed to assess compound toxicity against telomerase immortalized human gingival keratinocytes (TIGK) and the murine J774.A1 macrophage cell line. First, to determine whether compounds induce cell lysis or compromise cell membrane integrity, lactate dehydrogenase (LDH) activity released into the cell culture medium was measured after 18-h exposure of cells to each of the compounds. As shown in Fig. 4A, none of the compounds induced a significant increase in the release of LDH from TIGK cells over the range of concentrations that were tested. With J774A.1 cells, a small but statistically significant increase in LDH release was observed when cells were exposed to compound PCP-III-206 (40 μM), PCP-III-293 (60 μM), or PCP-IV-20 (60 μM) at the highest concentrations tested. However, under these conditions, the level of LDH release was still significantly less than the lysis control, suggesting that these three compounds may induce only low levels of cell lysis at high concentration. The effects of the compounds on TIGK and J774A.1 cells were also assessed by determining ATP levels, an indicator of metabolic activity. As shown in Fig. 5A, no significant reduction in ATP levels relative to the medium/dimethyl sulfoxide (DMSO) control was observed in TIGK cells, although the values obtained for compounds PCP-III-293 and PCP-IV-20 at 60 μM approached statistical significance. In contrast, treatment of J774A.1 cells with PCP-III-206 (at 20 μM and 40 μM) and PCP-IV-20 (at 60 μM) resulted in significant reductions in ATP levels (Fig. 5B), indicating that these compounds may impair the metabolic activity of J774A.1 cells. Interestingly, treatment of J774A.1 cells with compounds PCP-III-201, PCP-III-212, PCP-III-293, and PCP-IV-20 at 5 μM increased the levels of ATP (Fig. 5B), suggesting that these compounds at this concentration may stimulate metabolic activity of J774A.1 cells.

**FIG 6:**
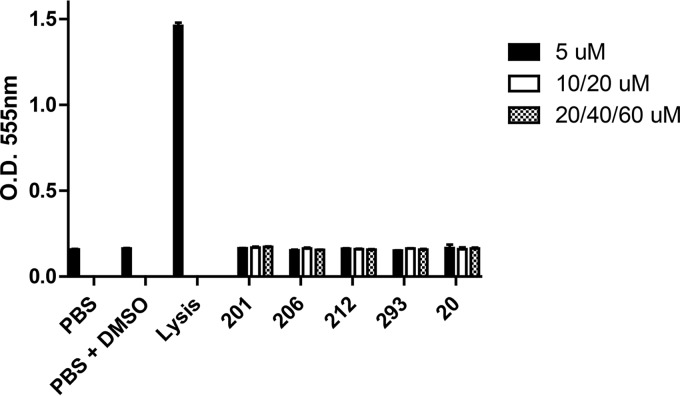
Hemolytic activity of peptidomimetic compounds against sheep red blood cells. The peptidomimetic compounds and concentrations used are as follows: PCP-III-201, 5 μM, 10 μM, and 20 μM; PCP-III-206, 5 μM, 20 μM, and 40 μM; PCP-III-212, 5 μM, 10 μM, and 20 μM; PCP-III-293, 5 μM, 20 μM, and 60 μM; PCP-IV-20, 5 μM, 20 μM, and 60 μM.

To determine whether exposure of cells to the peptidomimetic compound induced cell apoptosis, TIGK and J774A.1 cells were labeled with Sytox and Alexa Fluor 488-labeled annexin V and analyzed by flow cytometry. A representative series of images obtained from flow cytometry using TIGK cells is shown in Fig. S2, and a complete summary of the populations of live cells (Fig. S2, bottom left quadrant), early apoptosis (Fig. S2, bottom right quadrant), and late apoptosis/necrosis (Fig. S2, top right quadrant) for each cell line and compound tested is provided in Table 3. As shown, none of the compounds induced a significant increase in apoptotic cells relative to the control reactions for either of the cell lines, with the exception of TIGK cells exposed to PCP-III-293 at 60 μM. Finally, we also tested each of the compounds for hemolytic activity, and as shown in Fig. 6, none of the compounds induced hemolysis of sheep red blood cells. Similar results were observed using human red blood cells (data not shown). Together, these results indicate that the peptidomimetic compounds exhibited no toxicity toward human TIGK cells and minimal toxicity against J774A.1 cells at the concentrations that were previously shown to be effective in inhibiting P. gingivalis adherence to S. gordonii.

**TABLE 3:** TIGK and J774A.1 cell apoptosis induced by peptidomimetic compounds

Treatment	Concn (μM)	Live cells (%) (TIGK/J774A.1)	Early apoptosis (%) (TIGK/J774A.1)	Late apoptosis/necrosis (%) (TIGK/J774A.1)
Medium		91.8/96.7	2.1/0.4	0.9/0.6
Medium plus DMSO		96.9/97.2	1.6/0.3	0.6/0.3
H_2_O_2_ (5 mM)		50.0/21.3	25.6/7.1	10.9/21.4
PCP-III-201	5	94.7/97.4	0.9/0.3	0.7/0.5
	10	93.9/94.7	0.6/0.4	0.9/0.4
	20	95.7/93.9	0.9/0.5	0.9/0.6
PCP-III-206	5	96.9/97.1	0.9/0.3	0.5/0.5
	20	95.7/94.0	1.1/0.5	0.8/0.6
	40	95.9/89.7	0.7/0.4	0.6/0.6
PCP-III-212	5	95.0/98.4	0.7/0.2	2.3/0.1
	10	95.6/96.7	0.8/0.4	1.8/0.2
	20	95.3/94.8	0.8/0.2	1.9/0.2
PCP-III-293	5	92.2/93.2	1.2/0.5	4.4/0.2
	20	96.7/97.2	0.9/0.3	1.2/0.1
	60	78.3^*a*^/96.6	4.2/0.4	15.2*/0.2
PCP-IV-20	5	98.5/96.4	0.3/0.4	0.4/0.2
	20	94.4/96.0	1.1/0.3	3.2/0.1
	60	91.5/96.2	2.4/0.3	4.4/0.1

a *P* < 0.05.

## DISCUSSION

Interspecies adherence of P. gingivalis and commensal oral streptococci is important for the initial colonization of the oral cavity by P. gingivalis and thus represents a viable target for therapeutic intervention to limit pathogen colonization. Adherence is driven by a protein-protein interaction between the streptococcal antigen I/II polypeptide and the P. gingivalis minor fimbrial antigen (Mfa) and mediated by a discrete structural motif in antigen I/II (15, 24). Daep et al. showed that a synthetic peptide representing this motif functioned as a potent competitive inhibitor of adherence to streptococci and significantly reduced P. gingivalis virulence in a mouse model of periodontitis (16, 18). However, the use of peptides as therapeutic agents has limitations arising from the relatively high cost of peptide synthesis and their susceptibility to degradation by proteases expressed by oral organisms, including P. gingivalis itself. To address these limitations, Patil et al. designed and synthesized compounds that mimic the natural peptide substrate recognized by Mfa and identified several that were potent and stable inhibitors of P. gingivalis adherence to streptococci (19). The dental biofilm is very complex, and for these compounds to be effective in the oral cavity, they must be capable of reducing P. gingivalis adherence in the presence of other organisms that may independently interact with both P. gingivalis and streptococci. Our results show that the five compounds tested here prevent P. gingivalis biofilm formation even in the presence of F. nucleatum, a bridging organism that interacts with both P. gingivalis and streptococci independently of antigen I/II and Mfa. Furthermore, the compounds exhibit IC_50_s that are similar to those that were previously reported for inhibition of P. gingivalis adherence to streptococci, suggesting that the presence of the bridging organism does not substantially reduce their effectiveness. Under the conditions tested, the active compounds prevented the formation of the biofilm by inhibiting P. gingivalis adherence to streptococci. Thus, these compounds represent potential therapeutics that may be effective in preventing P. gingivalis colonization of the oral biofilm as it re-forms after a professional prophylaxis. However, compound PCP-III-201 also disrupted a preformed three-species biofilm, suggesting that active compounds may also potentially be effective in treating periodontitis by reducing biofilm load in the oral cavity. Unexpectedly, the compounds also inhibited the accumulation of F. nucleatum in a dose-dependent manner even though the mimetic compounds are specifically targeted to block the P. gingivalis/streptococcal interaction. One possible explanation for this is that the interaction of P. gingivalis with streptococci induces a complex adaptive response that involves changes in P. gingivalis gene expression and metabolic cross talk that promotes P. gingivalis biofilm formation and virulence (25–27). It is possible that this adaptive response in P. gingivalis may in turn also benefit other members of the microbial community, and thus, when P. gingivalis adherence to streptococci is prevented, the stability and persistence of the entire community are affected.

Four of the five compounds also significantly inhibited P. gingivalis-induced alveolar bone resorption in a mouse model of periodontitis. Bone loss that occurred in animals treated with the most potent compound, PCP-III-201, was not significantly greater than that in sham-infected mice. The remaining active compounds reduced bone loss but not to the extent of PCP-III-201, indicating that these compounds only partially inhibit P. gingivalis virulence. PCP-III-293 exhibited little effect on P. gingivalis virulence, which was unexpected, since this compound was a potent inhibitor of *in vitro* adherence to streptococci (19) and the formation of three-species biofilms. Given the gross structural similarities between the five inhibitors, such as the 2,4,5-trisubstituted oxazole, terminal aromatic groups, and the internal triazoles, the individual substituent atoms and overall molecular shape or conformation likely account for the differences in *in vivo* activity. The relationship of the aromatic methoxy groups of compound PCP-III-212 to the aromatic fluorines on PCP-III-201 is bioisosteric (28–30), but while the methoxynaphthalene rings common to PCP-III-212, PCP-III-293, and PCP-IV-20 represent a high degree of hydrophobicity, the steric demand of the naphthalenes is much greater than the *ortho*-methoxyphenyls of the more-potent PCP-III-201 and PCP-III-206. The most-active compounds represent compact molecules whereby the maximal intraatomic distances between the fluorine and methoxy groups are 18.5Å for PCP-III-201 and 18.4Å for PCP-III-206. In contrast, the other compounds assume more-bent conformations whereby the interatomic distances between each oxazole methoxy group and the naphthalenyl methoxy group are more variable (up to 24.9Å). Thus, the *in vivo* activity of the inhibitors appears to be favored by a more planar and more linear conformation with the halogenated aryl oxazoles bearing fluorine and bromine in both positions.

Since the peptidomimetic compounds were delivered simultaneously with P. gingivalis during the infection procedure, animals were only transiently exposed to the compounds. Under these conditions, none of the mice exhibited overt adverse reactions to the compounds. Furthermore, none of the compounds exhibited toxicity to human immortalized gingival keratinocytes. However, several compounds affected J774A.1 cells, albeit at relatively low levels and at the highest concentration tested. PCP-III-201 was not toxic to either cell type and was also the most potent inhibitor of P. gingivalis virulence, suggesting that it may represent a novel potential therapeutic agent that targets P. gingivalis and can serve as a lead compound to develop the next-generation inhibitors.

## MATERIALS AND METHODS

### Bacterial strains and culture conditions.

S. gordonii ATCC DL-1 was cultured in brain heart infusion broth (Difco) supplemented with 5% (wt/vol) yeast extract (BHIY) at 37°C for 16 h. P. gingivalis ATCC 33277 was grown in TSBY medium, which consists of 30 g/liter Trypticase soy broth (Difco) supplemented with 5% (wt/vol) yeast extract, 5 mg/liter hemin, and 1 mg/liter menadione under anaerobic conditions (10% CO_2_, 10% H_2_, and 80% N_2_) at 37°C for 48 h. Prior to inoculation, TSBY medium was reduced by incubation under anaerobic conditions for 24 h at 37°C. F. nucleatum ATCC 25586 was grown in reduced brain heart infusion (BHI) broth supplemented with 5 mg/liter hemin and 1 mg/liter menadione under anaerobic conditions (10% CO_2_, 10% H_2_, and 80% N_2_) at 37°C for 48 h.

### Formation of P. gingivalis biofilms.

To prepare bacterial cells for biofilm formation, 10 ml of an overnight S. gordonii culture was centrifuged at 3,700 × *g* for 5 min, and the cell pellet was suspended in 1 ml sterile phosphate-buffered saline (PBS) (10 mM Na_2_HPO_4_, 18 mM KH_2_PO_4_, 1.37 mM NaCl, and 2.7 mM KCl [pH 7.2]). Subsequently, 10 μl of a 5-mg/ml hexidium iodide solution (Molecular Probes) was added to the cell suspension and incubated for 15 min with gentle shaking at room temperature in the dark. The labeled cells were centrifuged as described above and washed with PBS, and the cell pellet was suspended in PBS at a final optical density at 600 nm (OD_600_) of 0.8. Similarly, 10 ml of 48-h cultures of P. gingivalis or F. nucleatum were centrifuged at 3,700 × *g* for 15 min, and the cell pellets were suspended in 1 ml reduced PBS. To label P. gingivalis or F. nucleatum, 20 μl of 5(6)-carboxyfluorescein *N*-hydroxysuccinimide ester (4 mg/ml; ThermoFisher) or 20 μl of 2 mM Cell Trace far red dye (DDAO-SE; ThermoFisher) was added to the cell suspension, respectively. After incubation for 30 min with gentle shaking at room temperature in the dark, each suspension was centrifuged, washed as described above, and suspended in PBS at a final OD_600_ of 0.4 for P. gingivalis or 2.0 for F. nucleatum. For biofilm formation, 1 ml of labeled S. gordonii cells was added to each well of a 12-well microtiter plate (Greiner Bio-one) containing a circular coverslip (ThermoFisher) and incubated under anaerobic conditions on a rotary shaker for 24 h at 37°C. Unbound cells were removed by aspiration, and 1 ml each of labeled P. gingivalis and F. nucleatum cells containing the desired concentration of test compound was added and incubated under anaerobic conditions for 24 h at 37°C. Test compounds were initially dissolved in dimethyl sulfoxide (DMSO) to generate 1,000× stock solutions and were routinely tested over a final concentration range of 0 to 60 μM. One microliter of the appropriate stock solution was added to each 1-ml aliquot of labeled P. gingivalis or F. nucleatum cells prior to adding the suspension to the microtiter plate wells. For control biofilms, 1 μl of DMSO was added to the *P. gingivalis/F. nucleatum* cell suspension and incubated as described above.

To test for activity against preformed biofilms, cells were grown and labeled as described above, and biofilms were formed in buffer alone for 24 h at 37°C. After removing unbound cells by aspiration, biofilms were incubated in 1 ml buffer containing the desired concentration of the test compounds and incubated for 1 to 3 h under anaerobic conditions.

### Visualization of *P. gingivalis-S. gordonii* biofilms.

To visualize P. gingivalis biofilms, unbound cells were removed by aspiration, and coverslips were washed once with PBS. Biofilms were fixed by incubating the coverslips with 1 ml of 4% paraformaldehyde for 5 min, followed by two washes with PBS. The coverslips were then removed, placed face down on a glass microscope slide containing a drop of Prolong Gold antifade reagent (Life Technology), and sealed with nail polish. Visualization of biofilms was conducted by laser scanning confocal microscopy with a Leica SP8 confocal microscope (Leica Microsystems Inc., Buffalo Grove, IL) using a 488-nm laser to detect labeled P. gingivalis, a 552-nm laser to detect S. gordonii, and a 633-nm laser to detect F. nucleatum. *Z*-plane scans of 25 μm in depth were collected at three randomly chosen frames on each coverslip using a z-step thickness of 0.7 μm. Background noise was minimized using software provided with the Leica SP8 microscope and three-dimensional reconstruction of the *Z*-plane scans and quantification of total green, red, and far red fluorescence was conducted using Volocity 6.3 image analysis software (PerkinElmer, Akron, Ohio). For biofilm images, far red fluorescence was arbitrarily assigned the color blue. The extent of P. gingivalis binding and accumulation was expressed as the ratio of total green (P. gingivalis) to red (S. gordonii) fluorescence, and the IC_50_ for each compound was defined as the concentration that reduced the ratio of green to red fluorescence by 50%. Experiments were carried out in triplicate for each concentration of test compound, and three independent experiments were conducted for each compound. GraphPad InStat3 software was used for data analysis, and statistical significance was defined as *P* < 0.05.

### *In vivo* model of periodontitis.

This protocol (protocol 16486) used for this study was approved on 3 May 2016 by the Institutional Animal Care and Use Committee at the University of Louisville as described in the federal guidelines for the care and use of laboratory animals. Specific-pathogen-free BALB/c/ByJ mice were obtained from The Jackson Laboratory (Bar Harbor, ME) when the mice were 10 weeks old and housed in the University of Louisville Research Resource Center animal facility. The mice were fed with Lab Diet 5001 meal form (Purina Mills, LLC, Gray Summit, MO) during the entire experiment.

Oral infection of mice was performed essentially as previously described by Daep et al. (18). S. gordonii was first established in the oral cavity of mice prior to infection with P. gingivalis. A total of seven mice per group were used per experiment. Animals were initially treated with a mixture of sulfamethoxazole (MP Biomedical, Solon, OH) at a final concentration of 800 μg/ml and trimethoprim (Sigma, St. Louis, MO) at a final concentration of 400 μg/ml *ad libitum* for 10 days. Four days after the last antibiotic treatment, the mice were orally infected with 10^9^ CFU S. gordonii cells suspended in 1 ml of 2% carboxymethyl cellulose (CMC; MP Biomedical, Solon, OH) in sterile PBS using a 2.25-mm feeding needle (Popper and Sons, Inc., New Hyde Park, NY). Subsequently, animals were infected five times with 10^7^ CFU P. gingivalis at 2-day intervals over a 10-day period. P. gingivalis cells were suspended in 1 ml of 2% CMC containing either the peptidomimetic compound or buffer. The concentrations of the peptidomimetic compounds administered were as follows: 20 μM for PCP-III-201, 40 μM for PCP-III-206, 20 μM for PCP-III-212, 15 μM for PCP-III-293, and 60 μM for PCP-IV-20; these concentrations were approximately threefold higher than the IC_50_ previously reported for inhibition of P. gingivalis adherence to S. gordonii (19). After infection, animals were allowed to rest for 47 days and then euthanized via CO_2_ asphyxiation. The total duration of the experiment was 80 days.

Mouse skulls were defleshed by autoclaving for 15 min, then immersed in 3% hydrogen peroxide overnight at room temperature to remove any remaining musculature, and washed with deionized water. The skulls were then soaked in 1% bleach solution for 30 s, sonicated at 14 V for 1 min, and then washed with water. To remove any remaining bacteria and tissues, the skulls were brushed with toothpaste and sonicated in 1% bleach solution for an additional 30 s at 14 V. The cleaned skulls were stained with 1% methylene blue for 15 s and rinsed with deionized water to remove excess dye. The stained skulls were allowed to air dry prior to measurement for alveolar bone loss.

Bone loss was assessed by measuring the distance between the alveolar bone crest (ABC) and the cemento-enamel junction (CEJ) at seven sites on the buccal side of the right and left maxillary molars for a total of 14 measurements. This was accomplished using a dissecting microscope fitted with a video imaging marker measurement system (model VIA-170K; Fryer) at a total magnification of ×40 (18). Measurements were taken in millimeters. The average of the total bone loss for each mouse group was assessed and subtracted from the baseline bone loss observed in sham-infected mice.

Statistical differences in bone loss were analyzed by analysis of variance (ANOVA) using GraphPad Instat (La Jolla, CA). A pairwise, parametric analysis of variance using a Bonferroni multiple-comparison posttest was used to determine the statistical difference among the individual mouse groups. A *P* value of ≤0.05 was considered to be statistically significant.

### Cell culture.

Human telomerase immortalized gingival keratinocytes (TIGKs) were provided by Richard Lamont (University of Louisville) and were authenticated by comparison to primary gingival epithelial cells for cell morphology, growth, cytokeratin expression, and the expression of Toll-like receptors. TIGKs were cultured at 37°C in an atmosphere of 5% CO_2_ in basal medium Dermalife K complete kit with Supplements (LifeLine, Frederick, MD). Cultures were incubated for 5 days and attained >95% confluence. The mouse macrophage cell line J774A.1 was obtained from the American Type Culture Collection and grown in Dulbecco modified Eagle medium (DMEM) (Thermo Fisher Scientific) supplemented with 4.5 g/ml glucose, 10% fetal bovine serum (FBS), and 100 U/ml penicillin-streptomycin (Sigma). Cultures were incubated at 37°C in an atmosphere of 5% CO_2_ for 4 days and reached >95% confluence.

### Measurement of LDH activity.

Lactate dehydrogenase (LDH) activity was determined using the CytoTox 96 nonradioactive cytotoxicity assay (Promega). TIGK and J774A.1 cells were inoculated in a 96-well microtiter plate at a density of 4,000 cells per well and grown for 24 h. The medium was then removed and replaced with fresh medium containing the desired concentration of peptidomimetic compound. The cells were further cultured for 18 h in the presence of the peptidomimetic compounds and centrifuged for 4 min at 250 × *g*, and 50 μl of supernatant was transferred to each well on a fresh 96-well microtiter plate. Subsequently, 50 μl of LDH substrate was added per well, and the plates were incubated at room temperature for 30 min. Reactions were terminated by the addition of 50 μl of stop solution provided in the CytoTox 96 kit. LDH activity was determined by measuring the optical density at a wavelength of 490 nm. For positive-control reactions, 15 μl of lysis buffer provided in the CytoTox 96 kit was added to the cells and incubated for 1 h. Negative-control reactions comprised cells that were incubated with medium alone. All samples were assayed in triplicate.

### Determination of cell metabolic activity.

Cell metabolic activity was assessed by quantifying total ATP levels in cell culture samples, using CellTiterGlo reagent (Promega). TIGK and J774A.1 cells were cultured and incubated with compounds as described above, washed three times with sterile PBS, and incubated with 100 μl of CellTiterGlo substrate for 2 min with shaking and for an additional 10 min without shaking. Total light production was measured using a Victor 3 multilabel plate reader (PerkinElmer) in luminometer mode. All samples were assayed in triplicate.

### Measurement of cell apoptosis.

The degree to which the peptidomimetic compounds induced apoptosis in TIGK and J774A.1 cells was determined using the phycoerythrin (PE) annexin V/dead cell apoptosis kit with SYTOX green for flow cytometry (Invitrogen). TIGK and J774A.1 cells were cultured in 12-well microtiter plates with an initial density of 2 × 10^5^ cells in 1.5 ml medium. After 24 h at 37°C, the medium was decanted, replaced with fresh medium containing the desired concentration of peptidomimetic compound, and incubated for an additional 18 h. The cells were washed with PBS, trypsinized, and centrifuged at 250 × *g*. The cell pellet was suspended in 100 μl of binding buffer supplemented with 1 μl Sytox and 5 μl annexin florescent dye and incubated for 15 min at 37°C. The Samples were then diluted by the addition of 400 μl binding buffer and analyzed by flow cytometry using a FACScalibur flow cytometer (Becton Dickinson), measuring the fluorescence emission at 530 nm and 575 nm. Medium only served as control.

### Determination of hemolytic activity.

A sample of 100 μl of 1% sheep or human erythrocytes (BioreclamationIVT, MD) was suspended in 1 ml sterile PBS containing 5% FBS, and the desired concentration of peptidomimetic compound was subsequently added. The suspension was incubated at 37°C for 3 h and centrifuged at 3,500 × *g* for 5 min, and 200 μl of the supernatant were transferred into each well of a 96-well microtiter plate. Hemoglobin release was recorded by spectrophotometry using a Victor 3 multilabel plate reader (PerkinElmer) at a wavelength of 538 nm. All samples were assayed in triplicate. Erythrocytes suspended in PBS with 5% FBS served as a negative control, and erythrocytes that were lysed by suspension in distilled water (dH_2_O) served as a positive control.

### Statistical analysis.

Data from each of the toxicity tests were analyzed by ANOVA using a Bonferroni multiple-comparison posttest to compare each experimental sample to the control sample. A *P* value of ≤0.05 was considered statistically significant.

## Supplementary Material

Supplemental material
