# Supplementary material for: In Vitro and In Vivo Activity of Peptidomimetic Compounds That Target the Periodontal Pathogen Porphyromonas gingivalis
Source: Antimicrob Agents Chemother. 2018 Jun 26;62(7):e00400-18. doi: 10.1128/AAC.00400-18 (PMC6021676; doi:10.1128/AAC.00400-18)
Supplement: Supplemental material [file supp_62_7_e00400-18__index.html]

Supplemental material 

# *In Vitro* and *In Vivo* Activity of Peptidomimetic Compounds That Target the Periodontal Pathogen Porphyromonas gingivalis

## Supplemental material

- Supplemental file 1 -

  Fig. S1 and S2

  PDF, 301K
