# Supplementary material for: In Vitro and In Vivo Activity of Peptidomimetic Compounds That Target the Periodontal Pathogen Porphyromonas gingivalis
Source: Antimicrob Agents Chemother. 2018 Jun 26;62(7):e00400-18. doi: 10.1128/AAC.00400-18 (PMC6021676; doi:10.1128/AAC.00400-18)

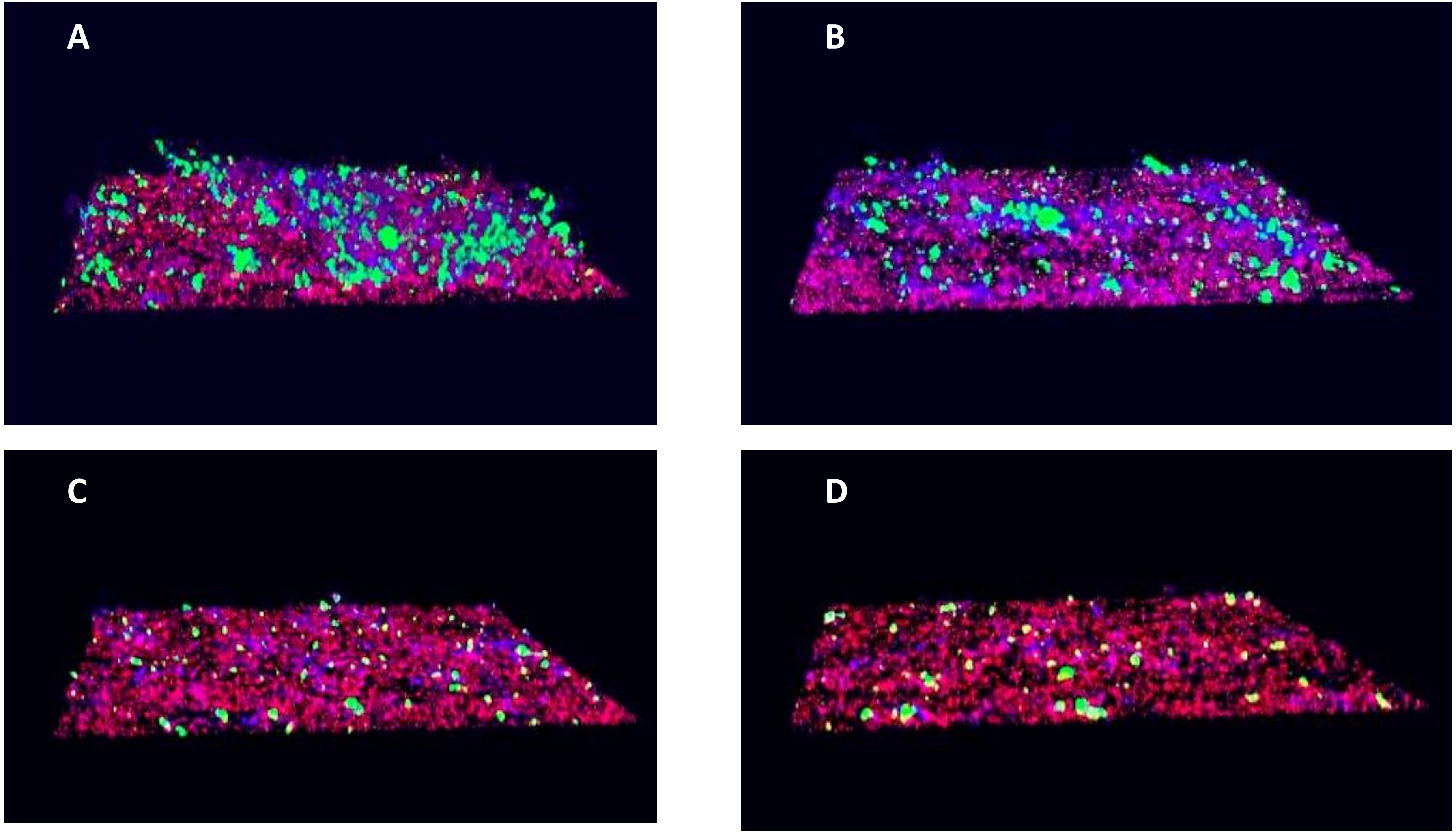

Figure S1. Inhibition of pre-formed biofilm by PCP-iii-201. Pre-formed three species biofilms were incubated in buffer alone (**A**) or buffer containing 5 μM (**B**), 10 μM (**C**) or 20 μM (**D**) PCP-iii-201 for 3 hrs were visualized by laser scanning confocal microscopy. Image stacks were assembled using Volocity Image Analysis software.

Figure S2. Representative flow cytometry profiles of TIGK cells after 18 hr exposure to peptidomimetic compounds. The lower left quadrant represents live cells, the lower right quadrant represents cells in early apoptosis and the upper right quadrant represents cells in late apoptosis or necrosis. Concentrations of compounds used is as follows: PCP-III-201 (20 μM), PCP-III-206 (40 μM), PCP-III-212 (20 μM), PCP-III-293 (60 μM), and PCP-IV-20 (60 μM).

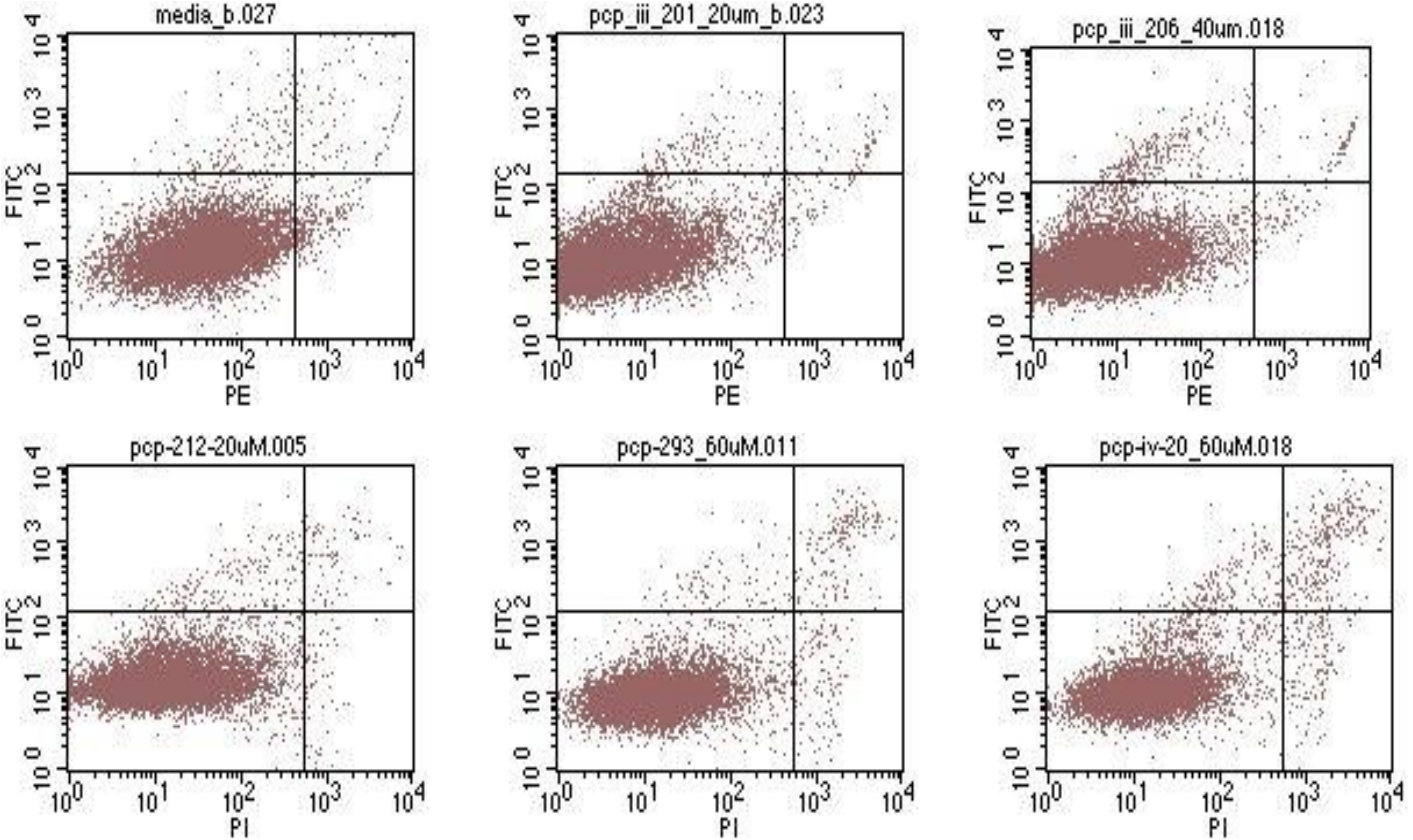

Supplement: Supplemental material [file AAC.00400-18_zac007187303s1.pdf]
